# External Employment of Liquor Chlorini

**Published:** 1850-11

**Authors:** 


*External Employment of Liquor Chlorini.*—DRS. CRAMER and SCHNEIDER have published communications recommending the more extensive use of chlorine as an external application. The good he had seen result from the application of *liquor chlorini* to malignant pustules and similar affections, induced Dr. Cramer to try its effects in bad furunculous swellings, the progress of which was thus surprisingly expedited, and the extension of the ulceration much limited, as compared to what occurs under the use of poultices. So, too, he has derived great advantage in employing it in large abscesses and in buboes, the matter sometimes becoming reabsorbed; and, where this is not so, the progress of the case is still very favorably influenced. Great relief followed its application to the neck, in a case of scarlatina, in a child, wherein suffocation seemed impending. He keeps compresses well soaked in the fluid constantly to the part. Dr. Schneider still more strongly recommends its use as a gargle, in variolous diseases, and in angina. He uses it diluted with water, and finds it exert a remarkable abortive power over variola, when affecting the tongue and throat, and angina in general.—*Brit. For. Med.-Chir. Rev.*, July, 1850, from *Casper's Wöchenschrift*, No. 8.

